# Anti-IgLON5 disease complicated with rectal adenocarcinoma: a case report

**DOI:** 10.1186/s12883-022-03044-y

**Published:** 2023-01-03

**Authors:** Yi Li, Lili Zhang, Yuzhong Wang

**Affiliations:** 1 Clinical Medical College, Jining Medical University, Jining, Shandong Province China; 2 Department of Neurology, Affiliated Hospital of Jining Medical University, Jining, Shandong Province China; 3 Medical Research Centre, Affiliated Hospital of Jining Medical University, Jining, Shandong Province China

**Keywords:** Autoimmune diseases, IgLON5, Rectal cancer, Movement disorders, Immunotherapy

## Abstract

**Background:**

Anti-IgLON5 disease is a rare chronic autoimmune-mediated tauopathy, featured by the sleep disturbance. Several studies have described its clinical characteristics, however, the simultaneous occurrence of anti-IgLON5 disease and rectal cancer has not been reported. We described an unusual entity of anti-IgLON5 disease complicated with rectal cancer.

**Case presentation:**

A 76-year-old man initially presented with slurred speech and limb tremors, followed by epileptic-like seizures, lethargy, and sleep apnea. IgG anti-IgLON5 antibodies were positive in both serum and cerebrospinal fluid. The patient responded well to the treatment of plasma exchange and a pulse and gradual reduction of steroids therapy. When the oral steroids started, mycophenolate mofetil was added. Two months later, the patient had bloody stools and pathological-confirmed moderately differentiated adenocarcinoma of the rectum.

**Conclusions:**

Whereas sleep disturbance is the most common feature in patients with anti-IgLON5 disease, our case presented with slurred speech and limb tremors. Anti-IgLON5 disease complicated with rectal cancer is very rare. Tumor screening should be considered in patients with anti-IgLON5 disease to investigate the association between tumor and this disease.

## Background

Anti-IgLON5 disease is a rare chronic autoimmune-mediated tauopathy mainly affecting aged people over 60 years old [1]. Its neuropathological features include gliosis, neuronal loss, and neuronal deposition of hyper-phosphorylated tau protein in specific areas such as the hypothalamus, brainstem tegmentum, etc [2]. The clinical symptoms and severity of anti-IgLON5 disease vary greatly among individuals, including sleep disturbance, gait disturbance, bulbar symptoms, disturbance of movement, and cognitive dysfunction [3]. Anti-IgLON5 disease complicated with malignant tumors is rarely reported till now. We herein described a patient with anti-IgLON5 disease complicated with rectal cancer who achieved relief of clinical symptoms after the plasma exchange and immunosuppression treatment.

## Case presentation

**Fig. 1:**
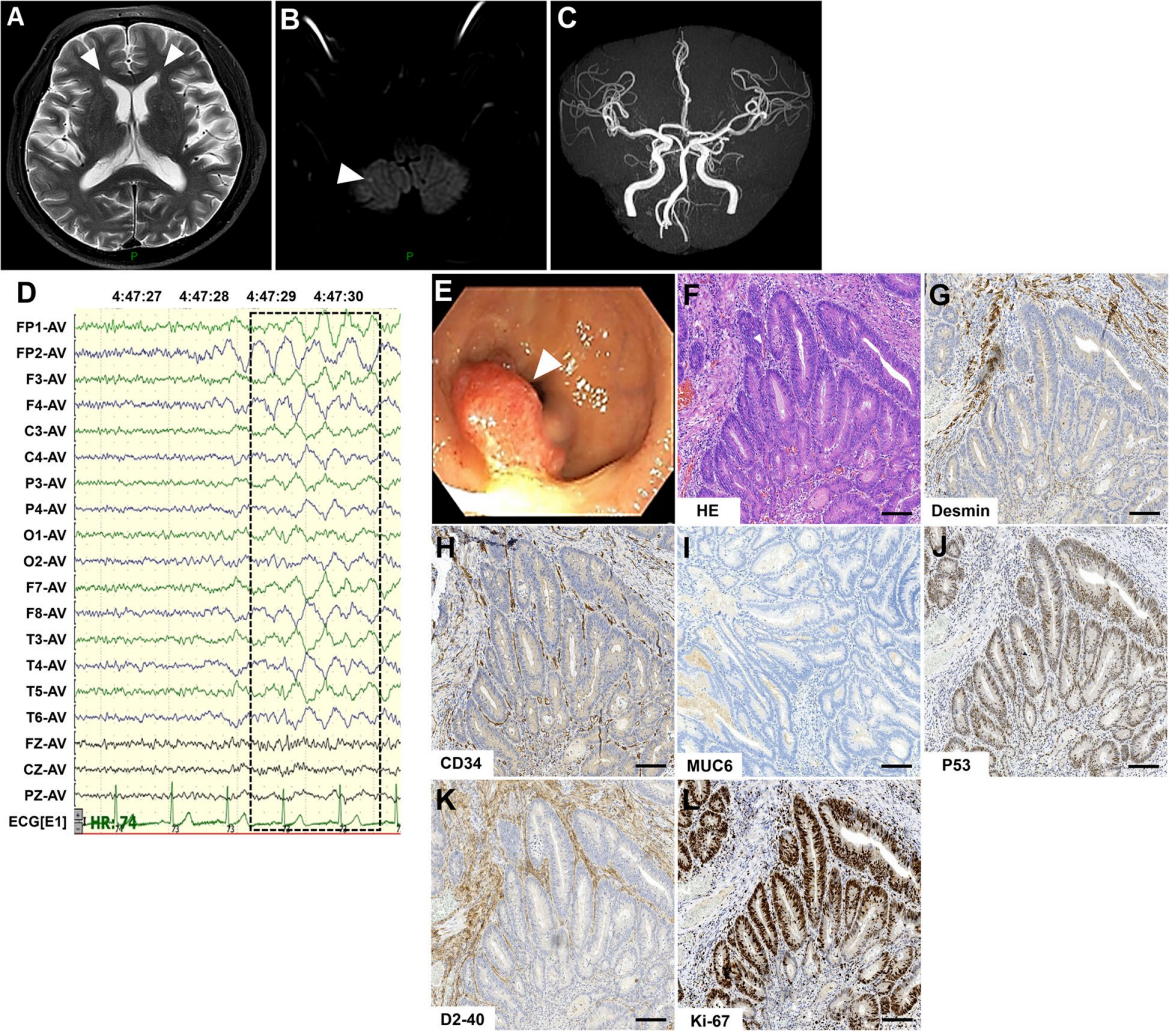
Magnetic resonance imaging, electroencephalogram and pathological findings. **A**, **B** and **C** Brain magnetic resonance imaging shows symmetric hyperdense on T2-weighted images around the anterior horn of the lateral ventricle and slightly restricted speckle diffusion signals in the right cerebellum on the diffusion-weighted image; angiography sequences showed spinal basilar artery tortuousness and mild cerebral arteriosclerosis. **D** Electroencephalogram shows that during the wakeful phase, moderate slow waves of about 2–2.5 HZ can be seen in each lead (frame), and no epilepsy waves are seen during the waking period and during sleep. **E** Colonoscopy shows that the rectum is about 6 cm from the anus to the entrance, and the surface is hyperemic and eroded. **F** Hematoxylin & eosin staining shows glandular fusion. Necrotic substances are seen in the glandular cavity and have infiltrated into the submucosal layer. Elongated or vacuole-like nuclei can be observed, as well as nuclear fission. **G**-**L** Immunohistochemistry shows positive for staining of Desmin, P53, Ki-67 (area for 40–50%), CD34 and D2–40. MUC6 staining is negative. Bar = 100μm

A 76-year-old male presented with 3 days of slurred speech and limb tremors in his extremities. He had a history of well-controlled hypertension for over a decade and no other documented medical history. The neurological signs at entry include lack of fluent speech, inability to perform rapidly alternating movements, and mild muscle tremors when his hands were raised horizontally. The renal function test showed a uric acid concentration of 533 μmol/L (202–416 μmol/L), and thyroid function tests exhibited an increased thyroid stimulating hormone concentration of 8.05mIU/L (0.27–4.2mIU/L). The other laboratory tests were all negative or normal, including blood routine test, blood lipid, and liver function, the spectrum of myocardial enzymes, B-type natriuretic peptide, erythrocyte sedimentation rate, urine routine test, glycosylated hemoglobins, coagulation function test, D-dimer determination, antinuclear antibodies, antineutrophil cytoplasmic antibodies, and cardiolipin antibodies. Infections of syphilis, human immunodeficiency virus, hepatitis B and C virus were all seronegative. Brain magnetic resonance imaging showed symmetric hyperdense on T2-weighted imaging around the anterior horn of the lateral ventricle (Fig. 1A), slightly restricted speckle diffusion signals in the right cerebellum on the diffusion-weighted imaging (Fig. 1B), and mild cranial arteriosclerosis (Fig. 1C). Chest computed tomography, electrocardiography, and cardiac color Doppler ultrasonography were almost normal. Based on the clinical signs and examination, he was diagnosed with ischemic cerebrovascular disease and stayed in-patient for observation.

On day three after admission, the patient developed an epileptic-like seizure while walking, presenting as loss of consciousness, limbs convulsion, eyes rolling, and foaming at the mouth for about 1 min. An urgent brain computed tomography showed no obvious signs of hemorrhage and infarction. A video electroencephalogram showed middle-amplitude slow waves at around 2–2.5 HZ in all leads during the awake period and no epileptic waves during both awake and sleep periods (Fig. 1D). Meanwhile, he developed symptoms of drowsiness, sleep apnea, and awakening from the airy obstruction. An electronic laryngoscope examination excludes the organic changes causing sleep apnea. Because the findings of brain magnetic resonance imaging and computed tomography could not explain the symptoms of seizure, drowsiness, and sleep disorders. A lumbar puncture was performed, and the intracranial pressure was normal (122.4 mmH2O). The cerebrospinal fluid analysis showed slightly increased protein levels of 0.47 g/L (reference value 0–0.4 g/L) and normal cell counts. An assay panel of autoimmune encephalitis and paraneoplastic syndrome, including autoantibodies against N-methyl-D-aspartate receptor, leucine-rich glioma inactivated 1, contactin-associated protein-like 2, metabotropic g-aminobutyric acid type B receptors, alpha-amino-3-hydroxy-5-methyl-4-isoxazolepropionic acid 1/2 receptor, IgLON5, dipeptidyl-peptidase-like protein 6, and glutamic acid decarboxylase and paraneoplastic-related antibodies (IgG anti-Hu, Yo, Ri, contactin response mediator protein 5, amphiphysin, Ma1, Ma2, SRY-box transcription factor 1, delta/notch-like epidermal growth factor-related receptor, Zic family member 4, protein kinase C gamma, Recoverin, and Titin antibodies) were implied (cell-based assay, Simcere co.,ltd, Nanjing, Jiangsu Province, China). He was positive for IgG anti-IgLON5 antibodies (1:1000 in the serum and 1:10 in the cerebrospinal fluid). A further human leukocyte antigen sequence-based typing detection revealed DQB1*04:01, DQB1*05:03, DRB1*04:05, and DRB1*14:05. The final diagnosis of anti-IgLON5 disease was made, and he was treated with plasma exchange (a total of two times every 2 days) and methylprednisolone pulse therapy (methylprednisolone sodium succinate, 1000 mg/day for 3 days, 500 mg/day for 3 days, 240 mg/day for 3 days, 120 mg/day for 3 days, and then 60 mg/day orally with a reduction of 5 mg every week till the end). Mycophenolate mofetil (0.5 g, twice daily) was added when the oral steroids started. Two weeks after immunotherapy, the patient showed obvious clinical improvement in speech, limb tremors, lethargy, and obstructive sleep apnea without epileptic seizures, and then he was discharged.

Two months later, the patient presented to the department of gastrointestinal surgery of our hospital because of unexplained bloody stools. The colonoscopic biopsy showed protrusion lesions in the lower rectum, with hyperemia and erosion on the surface (Fig. 1E). An endoscopic submucosal dissection was performed and the histopathological studies demonstrated the moderately-differentiated rectal adenocarcinoma (Fig. 1F), positive for staining of Desmin, P53, Ki-67 (area for 40–50%), CD34, and D2–40 but negative for MUC6 staining (Fig. 1G-L). Additionally, the methylation of the septin 9 gene is negative. He refused further radiotherapy and chemotherapy, then was discharged with oral steroids (gradual reduction) and mycophenolate mofetil therapy.

Six months after the first neurological signs, our patient continued the mycophenolate mofetil therapy. A follow-up showed that he still had intermittent bloody stool, mild sleepiness, sleep apnea, and awakening from the airy obstruction but no limb tremors, slurred speech, or epileptic-like seizures.

## Discussion and conclusions

Anti-IgLON5 disease can be a complex disorder to diagnose and its clinical presentation is heterogeneous and varies widely between patients. 57% (41/72) of the patients with anti-IgLON5 disease were initially diagnosed with movement disorders, among which the common abnormal movements consisted of gait and balance disorders (52 cases, 72%), chorea (24 cases, 33%), Bradykinesia (20 cases, 28%), dystonia (19 cases, 26%), abnormal body posture or rigidity (18 cases, 25%), and tremors (15 cases, 21%) [4]. Currently there are no definite diagnostic criteria for this disease, and detecting anti-IgLON5 antibodies in cerebrospinal fluid and serum is still a relatively recognized diagnostic basis. Our case presented to the hospital for slurred speech and limb tremors, which is a relative unspecific presentation of the anti-IgLON5 disease and increases the difficulty of rapid diagnosis. After the disease progressed gradually, the patient developed symptoms such as epileptic-like seizures and sleep apnea. The serum and cerebrospinal fluid were positive for anti-IgLON5 antibodies, which made a final diagnosis of anti-IgLON5 disease.

IgG anti-IgLON5 antibodies bound to the immunoglobulin-like domain 2 of the antigen led to irreversible antibody-mediated internalization of IgLON5 on the surface of cultured hippocampal neurons [5]. And thus, early diagnosis and immunotherapy to reduce the auto-antibodies-induced neuron damage are crucial for improving the prognosis of patients [6]. Brunetti et al. reported that the amelioration of neurological symptoms of patients with anti-IgLON5 disease may be related to the decrease of IgLON5 antibody titers [7]. Of the effective treatment for anti-IgLON5 disease, glucocorticoids were effective in 34% of patients (12/35), 43% (9/21) for intravenous immunoglobulin, 46% (7/15) for plasma exchange, 33% (4/12) for rituximab and 75% (3/4) for mycophenolate mofetil [8]. Gene polymorphisms of human leukocyte antigen (HLA) affect the response of patients to immunotherapy. Gaig et al. reported three out of five patients who were negative for HLA-DRB1*10:01 showed good response to immunotherapy (information of detailed treatment were not defined) while Cabezudo-García et al. summarized that all of the three patients who were positive for HLA-DQB1*05:01 but negative for HLA-DRB1*10:01 showed good response to treatment of intravenous immunoglobulin or intravenous immunoglobulin combined with steroids) [8, 9]. Our case who was negative for HLA-DRB1*10:01 and HLA-DQB1*05:01 showed good response to treatment of plasma exchange combined with steroids. These findings suggests that other genetic factors or different treatment may also affect the efficiency of immunotherapy for patients with anti-IgLON5 disease. Notably, current evidences for predicting the response of the patients to immunotherapy are mostly based on relatively small sample studies. A large-scale cohort study is necessary to investigate the factors predicting the efficiency of immunotherapy for the patients. He received plasma exchange to reduce antibody titers and methylprednisolone pulse therapy, followed by treatment of mycophenolate mofetil, which significantly improved the limb tremors and slurred speech but mild symptoms of sleep apnea and drowsiness remained.

Our case had confirmed moderately-differentiated rectal adenocarcinoma. A few patients were detected with carcinoma (adenocarcinoma of the breast) or had a history (prostate adenocarcinoma and non-Hodgkin lymphoma) [10], and Kaplan-Meier analysis showed higher expression of IgLON5 was significantly associated with shorter overall survival in patients with colon cancer [11]. Notably, a differential diagnosis of paraneoplastic syndrome should be excluded for patients with anti-IgLON5 disease and malignant tumors. Despite of the confirmed rectal adenocarcinoma, our case was seronegative for paraneoplastic syndrome-related antibodies and did not display the high-risk neurologic phenotypes [12], which do not support the diagnosis of paraneoplastic syndrome. Currently, the association between tumor and the production of anti-IgLON5 IgG antibodies as well as the anti-IgLON5 disease remains unknown. Our study had one limitation that the specimen of rectal adenocarcinoma was not available for us to further investigate the expression of IgLON5 protein in situ. More reports of the co-occurrence of anti-IgLON5 disease and tumor in patients would benefit further investigating the roles of tumor immunity in the action mechanism of IgG anti-IgLON5 antibody production and the pathogenesis of this disease.

Herein, we described an unusual entity with anti-IgLON5 disease complicated with rectal cancer. Despite the clinical signs of sleep apnea, our patient refused the polysomnogram, a very helpful evaluation for anti-IgLON5 disease, because of the economic reason. We recommend that clinicians screen patients with anti-IgLON5 disease for tumors in order to further explore the relationship between IgLON5 autoimmunity and tumors.

## Data Availability

Data are available from the author upon reasonable request and with permission of Affiliated Hospital of Jining Medical University.

## References

[1] Sabater L, Gaig C, Gelpi E, Bataller L, Lewerenz J, Torres-Vega E (2014). A novel non-rapid-eye movement and rapid-eye-movement parasomnia with sleep breathing disorder associated with antibodies to IgLON5: a case series, characterisation of the antigen, and post-mortem study. Lancet Neurol.

[2] Gelpi E, Höftberger R, Graus F, Ling H, Holton JL, Dawson T (2016). Neuropathological criteria of anti-IgLON5-related tauopathy. Acta Neuropathol.

[3] Gaig C, Graus F, Compta Y, Högl B, Bataller L, Brüggemann N (2017). Clinical manifestations of the anti-IgLON5 disease. Neurology..

[4] Gaig C, Compta Y, Heidbreder A, Marti MJ, Titulaer MJ, Crijnen Y (2021). Frequency and characterization of movement disorders in anti-IgLON5 disease. Neurology..

[5] Sabater L, Planagumà J, Dalmau J, Graus F (2016). Cellular investigations with human antibodies associated with the anti-IgLON5 syndrome. J Neuroinflammation.

[6] Grüter T, Behrendt V, Bien CI, Gold R, Ayzenberg I (2020). Early immunotherapy is highly effective in IgG1/IgG4 positive IgLON5 disease. J Neurol.

[7] Brunetti V, Della Marca G, Spagni G, Iorio R (2019). Immunotherapy improves sleep and cognitive impairment in anti-IgLON5 encephalopathy. Neurol Neuroimmunol Neuroinflamm.

[8] Cabezudo-García P, Mena-Vázquez N, Estivill Torrús G, Serrano-Castro P (2020). Response to immunotherapy in anti-IgLON5 disease: a systematic review. Acta Neurol Scand.

[9] Gaig C, Ercilla G, Daura X, Ezquerra M, Fernández-Santiago R, Palou E (2019). HLA and microtubule-associated protein tau H1 haplotype associations in anti-IgLON5 disease. Neurol Neuroimmunol Neuroinflamm.

[10] Honorat JA, Komorowski L, Josephs KA, Fechner K, St Louis EK, Hinson SR (2017). IgLON5 antibody: neurological accompaniments and outcomes in 20 patients. Neurol Neuroimmunol Neuroinflamm.

[11] Chen W, Huang J, Xiong J, Fu P, Chen C, Liu Y (2021). Identification of a tumor microenvironment-related gene signature indicative of disease prognosis and treatment response in Colon Cancer. Oxidative Med Cell Longev.

[12] Graus F, Vogrig A, Muñiz-Castrillo S, Antoine JG, Desestret V, Dubey D (2021). Updated diagnostic criteria for Paraneoplastic neurologic syndromes. Neurol Neuroimmunol Neuroinflamm.

